# Early urate-lowering therapy in gouty arthritis with acute flares: a double-blind placebo controlled clinical trial

**DOI:** 10.1186/s40001-022-00982-8

**Published:** 2023-01-06

**Authors:** Deng-Ho Yang, Hsiang-Cheng Chen, James Cheng-Chung Wei

**Affiliations:** 1 Division of Rheumatology/Immunology/Allergy, Department of Internal Medicine, Taichung Armed-Forces General Hospital, No 348, Sec. 2, Chung-Shan Rd, Taiping Dist., Taichung, 41152 Taiwan; 2 Department of Medical Laboratory Science and Biotechnology, Central Taiwan University of Science and Technology, Taichung, Taiwan; 3 Division of Rheumatology/Immunology/Allergy, Department of Internal Medicine, Tri-Service General Hospital, National Defense Medical Center, Taipei, Taiwan; 4 Institute of Biomedical Science, National Chung-Hsing University, Taichung, Taiwan; 5 Division of Allergy, Immunology and Rheumatology, Chung Shan Medical University Hospital, Taichung, Taiwan; 6 Institute of Medicine, Chung Shan Medical University, Taichung, Taiwan; 7 Graduate Institute of Integrated Medicine, China Medical University, Taichung, Taiwan

**Keywords:** Gout, Gouty arthritis, Uric acid, Probenecid, Urate-lowering therapy

## Abstract

**Background:**

Gouty arthritis (GA) is a chronic systemic disease with recurrent acute monoarthritis. In a previous study, a higher incidence of acute flares was observed during the initial marked decrease in serum urate level. Our study evaluated the effect of early urate-lowering therapy in patients with acute GA flares.

**Methods:**

This study included 40 patients with acute GA; of them, 20 received colchicine 0.5 mg colchicine twice daily, while 20 received probenecid 500 mg and colchicine 0.5 mg twice daily. We evaluated GA severity and laboratory data for 2 weeks after the initial therapy. Medians and interquartile ranges (IQRs) were calculated to evaluate clinical presentations between these two groups.

**Results:**

Rapidly decreasing median serum uric acid levels was found in the patients treated with probenecid and colchicine compared with the patients treated with colchicine alone on day 8 (− 1.9 [IQR, − 3.7 to 0] vs 0.8 [IQR, − 0.1–2.2]; P < 0.001). However, the median decrease in visual analog scale score did not differ significantly between the two groups (− 5.5 [IQR, − 8.0 to − 3.0] vs − 3.5 [IQR, − 5.9 to − 2.0]; P = 0.080).

**Conclusion:**

No significant increase was noted in acute gout flare severity or duration among GA patients treated with early aggressive control of hyperuricemia using probenecid plus colchicine.

## Introduction

Gout is a chronic systemic disease that features intermittent monoarthritis in different joints including the first metatarsal joint, tarsal joint, ankle, knee, and wrist. Most patients with gout may not take regular medication for its control; thus, intermittent arthritis attacks result. Except during recurrent arthritis episodes, systemic inflammation may develop during the progression of gouty arthritis (GA). Elevations in serum acute phase proteins, including C-reactive protein (CRP) and erythrocyte sedimentation rate (ESR), occur in patients with acute gout flare. Increased expression of different inflammatory cytokines, such as interleukin (IL)-6, IL-1, and tumor necrosis factor-α, are observed during acute gout attacks [1–3]. However, these cytokines are not measured in most patients clinically. Therefore, GA is not only an arthritis-related disease, it is a systemic inflammatory disease during acute flares. Clinically higher rates of cardiovascular events are observed in patients with GA than in the general population. Further, hyperuricemia is always found in patients with GA. Hyperuricemia is associated with a higher body mass index, blood pressure, and hyperlipidemia [4]. Recurrent arthritis in GA patients may induce tophus formation and erosive joint damage if GA remains uncontrolled. Aggressive formation of multiple tophi in polyarthritis may also lead to the progression to end-stage GA with significant joint deformity. Chronic kidney disease and intermittent formation of renal stone-related nephrolithiasis commonly occur in patients with gout [5]. Patients with GA have different co-morbidities, including hypertension, diabetes, and chronic kidney disease. In the management of GA, the long-term use of nonsteroidal anti-inflammatory drugs (NSAIDs) may enhance the progression of organic damage including renal, peptic ulcer, and cardiovascular diseases. Therefore, urate-lowering therapy (ULT) is important for GA evaluations and observations. Inhibiting urate production and stimulating urate renal secretion are the two major mechanisms of action of ULT drugs. These uricosuric agents include benzbromarone, probenecid, and sulphinpyrazone, while xanthine oxidase inhibitors include allopurinol and febuxostat. After ULT, a patient’s serum uric acid level may be decreased. Previous studies reported that ULT therapy was associated with a lower risk of cardiovascular disease and mortality [6, 7]. However, no randomized controlled trials to date have demonstrated a direct link between hyperuricemia and cardiovascular disease. In chronic GA, aggressive control of hyperuricemia may reduce tophus formation [8]. Oral colchicine, NSAIDs, oral steroids, and intra-articular steroid injections are used to manage acute gouty arthritis attacks [9]. Quick increases or decreases in serum uric acid levels by dietary or lifestyle risk factors can induce acute gout flare [10]. In a previous clinical trial, a higher incidence of acute gout flare was observed during the initial marked decrease in serum urate levels [11]. The incidence of acute gout flare gradually decreases after adequate ULT medication. Initiating ULT may increase the risk for acute gout attack. Should early ULT be started during acute flares in GA patients or not until they resolve? Our study evaluated the effect of early probenecid administration in patients with acute gout flares.

## Methods

### Participants

**Fig. 1:**
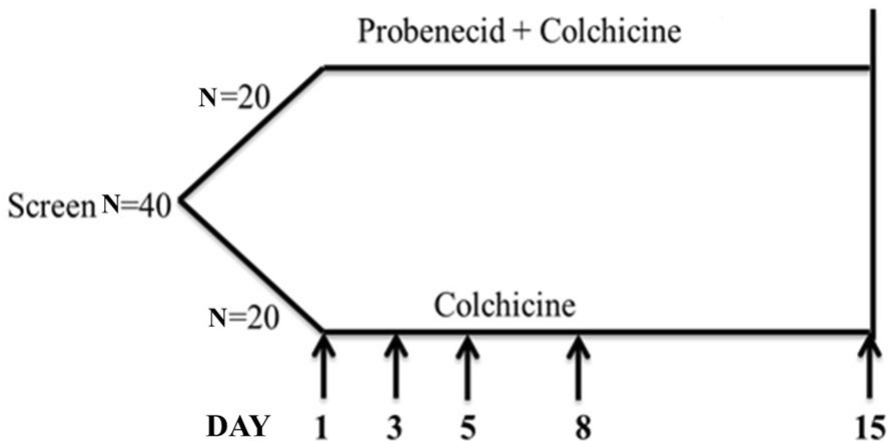
Experimental design. Patients with GA were evaluated in the clinic on days 1, 8, and 15. On days 3 and 5, the arthritis frequency, medication-related dose, and pain severity were evaluated via telephone

This study included 40 patients with an acute gout attack (within 72 h) who received adequate medication. The patients were initially diagnosed with gout in the clinical setting. The diagnosis of GA was made according to the 1977 American Rheumatism Association preliminary criteria for gout [12]. The study was approved by the Institutional Review Board of Tri-Service General Hospital (TSGHIRB No. 1-104-05-001), and all patients provided written informed consent. These patients were divided randomly into two groups via a random number table. The control group included 20 patients receiving colchicine 0.5 mg twice daily. The treatment group included 20 patients receiving probenecid 500 mg and colchicine 0.5 mg twice daily. Hybrid pills of colchicine and probenecid were given to the patient in the treatment group, and pills of colchicine were given to the patients in control group. These participants did not know to which of those two groups they were assigned. The researchers and evaluator conducting this study also did not know to which group the participants had been assigned. For two weeks (days 1–15), the patients were allowed to use aceclofenac 100 mg intermittently to control GA-related pain. We excluded patients with NSAID allergy, renal insufficiency (estimated glomerular filtration rate, < 30 mL/min), and hepatic insufficiency (glutamic pyruvic transaminase [GPT], > 120 U/mL); those receiving anti-tuberculosis drugs; and those who were pregnant. The duration of medication usage was 15 days. The patients underwent a physical examination, visual analog scale (VAS) pain scoring, and blood tests. We evaluated the GA-related pain severity and duration, and the primary endpoint was the intergroup difference in VAS scores on day 8. Patients with GA were evaluated in the clinic on days 1, 8, and 15. On days 3 and 5, the arthritis frequency, medication-related dose, and pain severity were evaluated via telephone. The laboratory test included uric acid, complete blood count, creatinine, GPT, and CRP measurements. The study design is shown in Fig. 1.

### Sample size estimation

This was a non-inferiority trial. The definition of non-inferiority margin was 1.3 and the desirable significant level α was 5%. The value of Power 1-β was 80%. The main treatment effect was measured by the VAS difference at days 1 and 8. We expected decreases from 8 points to 4.5 points in the treatment group and from 8 points to 3.2 points in the control group after treatment. The standard deviation was 1.5. The random allocation rate was 1 to 1 and the expected dropout rate was 20%. Finally, a sample size of 40 participants with complete data was required.

### Statistical analysis

All statistical analyses were conducted using SPSS software (version 15.0; SPSS Inc., Chicago, IL, USA), and values of *p* < 0.05 were considered statistically significant. Numeric variables were presented as mean values with standard deviations, median values with interquartile range (IQR). Numeric variables include age, body height, body weight, body mass index, systolic blood pressure, diastolic blood pressure, white blood cell count, red blood cell count, hemoglobin, hematocrit, mean corpuscular volume, mean corpuscular hemoglobin, platelet count, GPT, creatine, CRP, uric acid, and VAS scores. The Mann–Whitney U test was used to compare patients’ difference among the study groups, including the baseline demographics statistical differences and presentations by group on days 8 and 15. Two-tailed tests were performed for each scenario. The 95% confidence interval (95% CI) was used for mean VAS score, mean uric acid level, and average days of acute gout-related pain. Finally, the Generalized Estimating Equation (GEE) was applied to analyze the VAS change after adjusting for the study groups and frequency medication of aceclofenac.

## Results

### Basic demographic and clinical characteristics of the study cohort

**Fig. 2:**
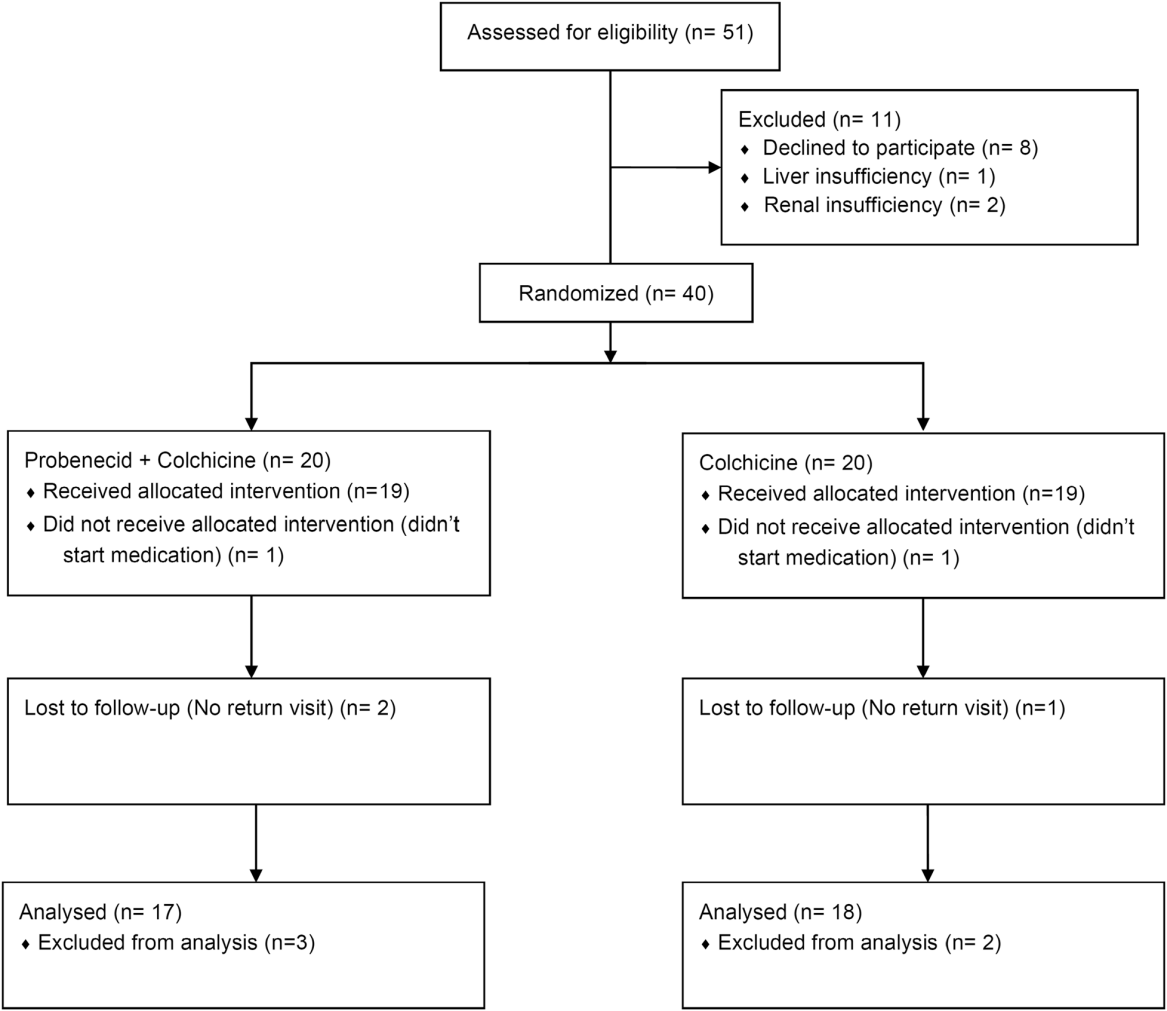
Flow diagram of subject enrollment process

In this open randomized controlled trial, the baseline characteristics were similar between the two groups (Table 1). Forty patients were randomly assigned to two groups: 20 receiving probenecid and colchicine twice daily and 20 receiving only colchicine twice daily. No significant intergroup differences were noted in age, sex, body mass index, blood pressure, hemoglobin, liver function, or renal function. Initial serum biomarkers of leukocytosis, platelet count, and CRP were similar. VAS pain scores did not differ between the two groups. In the treatment group, 17 patients completed the full course of the study (two were lost to follow-up; one refused therapy). In the control group, 18 patients completed the full course of the study (one was lost to follow-up and one refused therapy). We used intention to treat to evaluate missing data in this study. A study flow diagram is shown in Fig. 2.

**Table 1:** Patients’ baseline demographics by study group

	All *n* = 40	Treatment group *n* = 20	Control group *n* = 20	*P* value
	Median (IQR)	Median (IQR)	Median (IQR)	
Sex (male/female)		19/1	19/1	
Age (years)	38.0(30.0–47.5)	34.5(29.3–43.0)	39.0(31.5–49.0)	0.198
Body height (cm)	170.50(166.0–175.8)	171.0(168.0–175.0)	169.0(165.0–178.8)	0.655
Body weight (kg)	76.2(68.5–90.0)	76.2(72.3–88.3)	76.0(65.8–91.5)	0.797
BMI (kg/m^2^)	26.4(24.2–29.1)	26.5(24.2–29.0)	26.4(23.8–29.3)	0.978
SBP (mmHg)	130.0(122.3–139.8)	126.0(121.3–136.8)	132.5(126.5–141.5)	0.163
DBP (mmHg)	84.0(72.8–88.0)	81.0(70.3–88.0)	85.0(79.3–88.8)	0.302
WBC (/μl)	8725(7125–10275)	8910(7515–10115)	8725(6622–10525)	0.776
RBC (10^4^/μl)	520.5(480–535)	525.0(491.3–556.5)	501.0(468.3–528.8)	0.079
Hb (g/dl)	14.9(14.3–15.7)	15.1(14.0–16.1)	14.8(14.3–15.7)	0.871
Hct (%)	44.0(41.8–45.8)	43.9(41.5–46.6)	44.1(42.6–45.7)	0.685
MCV (fl)	87.0(84.3–90.3)	85.8(83.4–87.6)	87.8(85.7–92.0)	0.022
MCH (Pg)	29.4(29.0–30.7)	29.4(28.8–30.1)	30.4(29.1–31.0)	0.104
MCHC (g/dl)	33.9(33.5–34.5)	34.2(33.5–34.8)	33.7(33.5–34.3)	0.316
Platelet (/μl)	236.5 K(211 K–267.5 K)	243.5 K(205 K–261.5 K)	234.5 K(212 K–269 K)	0.756
GPT (IU/l)	29.5(17.0–41.8)	31.0(13.8–37.0)	28.0(17.0–43.8)	0.498
Creatine (mg/dl)	1.00(0.9–1.1)	1.0(0.9–1.1)	0.9(0.8–1.0)	0.182
CRP (mg/dl)	0.6(0.2–1.7)	0.5(0.2–1.3)	0.7(0.5–1.9)	0.351
Uric acid (mg/dl)	7.3(5.9–8.3)	7.3(5.9–8.3)	7.4(5.9–8.4)	0.892
VAS	5.0(3.0–8.0)	5.5(3.6, 9.0)	5.0(3.0–7.0)	0.241

*BMI* body mass index, *CRP* C-reactive protein, *DBP* diastolic blood pressure, *GPT* glutamic pyruvic transaminase, *Hb* hemoglobin, *Hct* hematocrit, *MCV* mean corpuscular volume, *MCH* mean corpuscular hemoglobin, *RBC* red blood cell, *SBP* systolic blood pressure, *WBC* white blood cell

^a^Mann–Whitney U test for the difference compared between the treatment group and control group

^*^P < 0.05

### No significant intergroup difference in decreasing VAS scores on day 8

A rapid decrease in serum uric acid level was identified in the patient group treated with combination probenecid and colchicine versus that in the group treated with colchicine only on day 8 (− 1.9 [IQR, − 3.7 to 0] vs 0.8 [IQR, − 0.1–2.2]; *P* < 0.001). In the control group, the levels of uric acid remained persistently high. However, the median decrease in VAS score did not differ between groups on day 8 (− 5.5 [IQR, − 8.0 to − 3.0] vs − 3.5 [IQR, − 5.9 to − 2.0]; *P* = 0.080). Serum CRP levels were also adequately controlled in the two groups (− 0.2 [IQR, − 0.9 to 0] vs − 0.4 [IQR, − 1.1 to − 0.1]; *P* = 0.336). A significantly smaller increase in median GPT level was identified in the treatment versus control group (0 [IQR, − 3.8 to 7.3] vs 4.5 [IQR, − 1.8 to 26.8]; *P* = 0.107). No significant intergroup difference in complete blood count and creatine findings was observed on day 8. The differences between the treatment and control groups on day 8 are shown in Table 2.

**Table 2:** Patient presentations by group on days 8 and 15

	Treatment group (D8) *n* = 20		Control group (D8) *n* = 20		*p*-valuea
	Median (IQR)	Mean ± SD	Median (IQR)	Mean ± SD	
Body weight (kg)	0.0(0.0 to 0.0)	0.25 ± 0.64	0.0(0.0 to 0.0)	0.28 ± 1.14	0.433
SBP (mmHg)	1.00(− 10.0 to 7.8)	0.60 ± 13.11	1.0(− 7.8 to 7.3)	2.30 ± 11.98	0.745
DBP (mmHg)	0.00(− 5.8 to 3.5)	0.40 ± 8.31	0.0(− 7.5 to 6.0)	1.80 ± 14.62	0.903
WBC (/μl)	− 960(− 2622.5 to − 55.0)	− 1392 ± 1505	− 1120(− 2642.5 to − 177.5)	− 1368.5 ± 1774.3	0.903
RBC (104/μl)	6.0(0.0–20.3)	10.30 ± 19.02	− 4.0(− 11.3 to 9.3)	− 0.25 ± 18.31	0.042*
Hb (g/dl)	0.0(− 0.1 to 0.7)	0.31 ± 0.67	− 0.1(− 0.4 to 0.4)	− 0.02 ± 0.52	0.139
Hct (%)	0.5(− 0.2 to 2.0)	0.88 ± 1.68	− 0.2(− 1.6 to 1.0)	−0.26 ± 1.78	0.047*
MCV (fl)	0.0(− 0.4 to 0.5)	0.01 ± 0.64	− 0.3(− 1.0 to 0.3)	− 0.42 ± 0.81	0.143
MCH (Pg)	0.0(− 0.4 to 0.3)	− 0.02 ± 0.44	− 0.1(− 0.5 to 0.4)	− 0.01 ± 0.46	0.968
MCHC (g/dl)	0.0(− 0.4 to 0.4)	− 0.03 ± 0.44	0.1(− 0.2 to 0.6)	0.13 ± 0.53	0.408
Platelet (/μl)	11.5 K(− 6.5 K to 27.5 K)	10750 ± 26098.7	8 K(− 6.5 K to 25.3 K)	− 900 ± 47901.1	0.787
GPT (IU/l)	0.0(− 3.8 to 7.3)	1.15 ± 7.68	4.5(− 1.8 to 26.8)	11.40 ± 18.36	0.107
Creatine (mg/dl)	0.1(0.0 to 0.2)	0.07 ± 0.12	0.02(0.0 to 0.1)	0.06 ± 0.12	0.494
CRP (mg/dl)	− 0.2(− 0.9 to 0.0)	− 0.55 ± 1.11	− 0.4(− 1 to − 0.1)	− 1.00 ± 1.96	0.336
Uric acid (mg/dl)	− 1.9(− 3.7 to 0.0)	− 1.77 ± 2.037	0.8(− 0.1 to 2.2)	1.24 ± 2.084	< 0.001***
VAS	− 5.5(− 8.0 to − 3.0)	− 5.2 ± 3.09	− 3.5(− 5.9 to − 2.0)	− 3.63 ± 2.38	0.080

	Treatment group (D15) *n* = 20		Control group (D15) *n* = 20		*p*-valuea
	Median (IQR)	Mean ± SD	Median (IQR)	Mean ± SD	
Body weight (kg)	0.0(0.0 to 0.0)	0.20 ± 0.70	0.0(0.0 to 0.0)	0.06 ± 0.34	0.638
SBP (mmHg)	0.5(− 6.8 to 7.8)	− 0.25 ± 9.78	2.5(− 13.3 to 5.5)	− 1.30 ± 13.09	0.892
DBP (mmHg)	− 0.5(− 6.0 to 5.3)	− 1.15 ± 11.39	0.0(− 12.3 to 5.5)	− 1.85 ± 11.53	0.946
WBC (μl)	− 1115(− 2805 to 0.0)	− 1394 ± 1820.5	− 1200(− 2682.5 to − 225.0)	− 1266.5 ± 2261.4	0.892
RBC (10^4^/μl)	2.0(− 17.8 to 18.5)	1.45 ± 19.69	4.0(− 10.0 to 11.5)	2.20 ± 18.02	0.946
Hb (g/dl)	0.1(− 0.6 to 0.5)	0.01 ± 0.57	0.1(− 2.0 to 0.4)	− 0.02 ± 0.52	0.870
Hct (%)	0.0(− 1.7 to 1.5)	0.05 ± 1.56	0.1(− 1.3 to 1.2)	− 0.07 ± 1.78	0.989
MCV (fl)	− 0.1(− 0.4 to 0.1)	– 0.23 ± 0.50	− 0.8(− 1.0 to 0.4)	− 0.49 ± 1.06	0.316
MCH (Pg)	− 0.1(− 0.4 to 0.3)	− 0.11 ± 0.44	–0 .1(− 0.4 to 0.1)	− 0.15 ± 045	0.786
MCHC (g/dl)	0.0(–0 .3 to 0.4)	− 0.02 ± 0.44	− 0.1(− 0.3 to 0.3)	− 0.01 ± 0.59	0.645
Platelet (μl)	8 K(− 22.3 K–27.8 K)	5050 ± 28943.6	2 K(− 8.3 K–21.5 K)	− 16800 ± 76110.4	0.579
GPT (IU/l)	0.0(− 2.0 to 4.8)	1.20 ± 5.90	7.0(− 0.8 to 15.5)	10.50 ± 17.77	0.029*
Creatine (mg/dl)	0.0(0.0 to 0.1)	0.02 ± 0.14	0.0(− 0.1 to 0.1)	0.04 ± 0.11	0.741
CRP (mg/dl)	− 0.3(− 1.1 to 0.0)	− 0.89 ± 1.53	− 0.5(− 1.4 to − 0.2)	− 01.12 ± 2.02	0.213
Uric acid (mg/dl)	− 1.7(− 2.9 to 0.0)	− 1.36 ± 1.82	0.3(− 0.2 to 1.0)	0.81 ± 2.09	0.002**
VAS	− 5.5(− 8.0 to − 3.1)	− 5.68 ± 3.01	− 4.8(− 7.0 to − 2.0)	− 4.37 ± 2.86	0.161

*BMI* body mass index, *CRP* C-reactive protein, *DBP* diastolic blood pressure, *GPT* glutamic pyruvic transaminase, *Hb* hemoglobin, *Hct* hematocrit, *MCH* mean corpuscular hemoglobin, *MCV* mean corpuscular volume, *RBC* red blood cell, *SBP* systolic blood pressure, *WBC* white blood cell, *IQR* Interquartile range

^a^Mann–Whitney U test for the difference compared between the treatment group and control group

^*^P < 0.05, **P < 0.01, ***P < 0.001

### No significant difference in GA-related pain noted between treatment and control groups on day 15

After 2 weeks of therapy, a persistent decrease in serum CRP levels was observed in the two groups (− 0.3 [IQR, − 1.1 to 0] vs − 0.5 [IQR, − 1.4 to − 0.2]; *P* = 0.213). There was no significant intergroup difference in improvement of VAS scores (− 5.5 [IQR, − 8.0 to − 3.1] vs − 4.8 [IQR, − 7.0 to − 2.0]; *P* = 0.161). A significant decrease in serum uric acid levels was still observed in the treatment versus control group on day 15 (− 1.7 [IQR, − 2.9 to 0] vs 0.3 [IQR, − 0.2 to 1.0]; *P* = 0.002). Remarkably, maintained liver function (GPT) without elevations was observed in the treatment versus control group (0 [IQR, − 2.0 to 4.8] vs 7.0 [IQR, − 0.8 to 15.5]; *P* = 0.029). There was no significant intergroup difference in the other laboratory finding including complete blood count, creatine, and CRP on day 15. The differences between the treatment and control group on day 15 are shown in Table 2.

### Early lowering of serum uric acid levels could not activate acute GA pain under colchicine administration

**Fig. 3:**
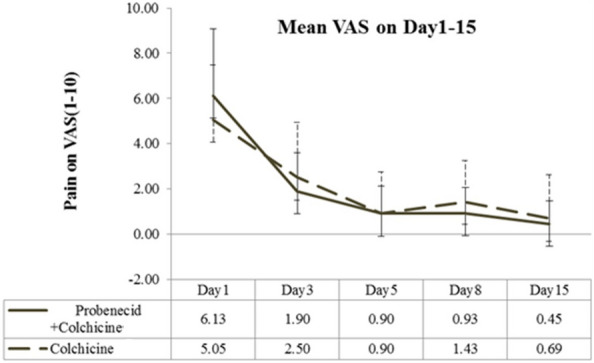
Mean visual analog scale (VAS) score over study period. Error bars represent 95% confidence intervals

**Fig. 4:**
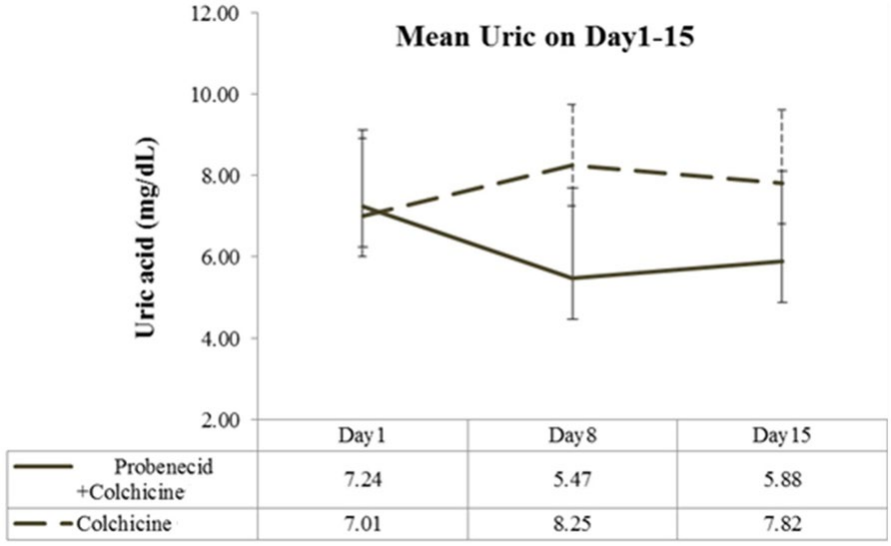
Mean uric acid level over study period. Error bars represent 95% confidence intervals

**Fig. 5:**
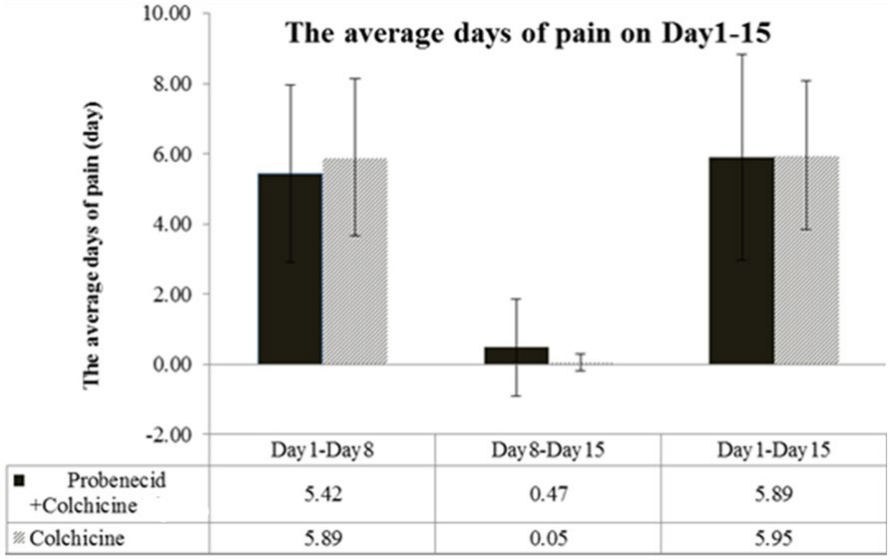
Mean pain duration over study period. Error bars represent 95% confidence intervals

From days 1–15, pain severity (VAS score) was similar in the treatment and control groups (Fig. 3). A significantly decreasing VAS score was observed on day 3 and a persistent low VAS was noted on days 5, 8, and 15 in both groups. No significant intergroup difference was detected in the progressive decrease evidenced by VAS pain scores. A significant decrease in the serum uric acid levels was observed in the treatment group, while persistently high levels of uric acid were observed in the control group (Fig. 4). The average number of days of GA-induced pain did not differ between groups (Fig. 5). Overall, during days 1–15, the average number of aceclofenac pills used for intermittent pain control did not differ significantly between groups (5.8 vs 6.05; *P* = 0.907). We used GEE to evaluate the different presentations of VAS in these two groups. In model I, there was no significant difference of VAS scores between control group and treatment group (*P* = 0.828). We added the factor of used aceclofenac pills in the Model II. There was no significant difference of VAS scores between control group and treatment group (*P* = 0.786). The change of VAS score by GEE is shown as Table 3.

**Table 3:** The change of VAS score by generalized estimating equations

	Model I		Model II	
	β(95%CI)	*p*	β(95%CI)	*p*
Group				
Control	Ref.		Ref.	
Treatment	− 0.123(− 1.237, 0.991)	0.828	− 0.310(− 1.348, 0.729)	0.559
Used aceclofenac			− 0.029(− 0.238, 0.180)	0.786

^*^P < 0.05, **P < 0.01, ***P < 0.001

## Discussion

In this open randomized controlled trial, the baseline characteristics were similar in the two groups. Significant decreasing levels of serum uric acid were found in the treatment group. No significant increase in pain severity or duration was observed in the treatment versus control group.

Treatment for GA includes the management of acute gout flares, prevention of recurrent arthritis, and control of hyperuricemia. The first-line medications for acute gout flares are oral colchicine, NSAIDs, and corticosteroids. After the control of acute gout flares is achieved, xanthine oxidase inhibitors and uricosuric agents can be used as ULT. Allopurinol can be used for the initial management of hyperuricemia in patients with chronic kidney disease [13]. The clinical response to allopurinol is similar to that of uricosuric agents; however, febuxostat induces a steeper effect on serum urate reduction than allopurinol [14]. Colchicine may inhibit monosodium urate-induced superoxide production by human neutrophils in vitro and effectively prevents acute gout flares [15, 16]. Probenecid may be used as monotherapy or in combination with allopurinol and still effectively lower hyperuricemia in GA [17].

Different ULT drugs including allopurinol, febuxostat, pegloticase, benzbromarone, and probenecid significantly lower serum urate levels compared with placebo [18]. Among these uricosuric agents, benzbromarone has greater efficacy at achieving serum urate normalization than probenecid [19]. Colchicine, low-dose NSAIDs, and low-dose steroids are recommended as ULT for the prophylaxis of acute gout attacks [20]. However, no strong clinical trial to date has proven the prophylactic effect of colchicine. Our study showed a rapid decrease in serum uric acid levels in the patient group treated with probenecid and colchicine compared with the group treated with only colchicine alone on day 8 (Table 2). After one week of medication, significantly improved pain was reported by the two groups. The mean VAS was not significantly different between the two groups (− 5.5 [IQR, − 8.0 to − 3.0] vs − 3.5 [IQR, − 5.9 to − 2.0]; *P* = 0.080).

Serum CRP levels were also adequately controlled in the two groups. Early intervention using ULT to induce the aggressive control of serum uric acid levels could have achieved early hyperuricemia control but no development of poor control of GA-related pain. The prophylactic effect of acute gout flare treatment may be due to the effect of colchicine and NSAIDs. Continued treatment with low-dose colchicine may inhibit monosodium urate-induced superoxide production by neutrophils in vivo [10]. After 2 weeks, a persistent decrease in serum CRP levels was observed in the two groups (Table 2). No significant intergroup difference in VAS score improvement was noted. According to a previous review, the early initiation of allopurinol does not increase pain severity or acute gout attack [21]. Therefore, the early intervention of hyperuricemia by probenecid using colchicine could prevent GA with acute flares.

Probenecid treats GA by inhibiting uric acid reuptake in the renal tubular transporter. Multiple effects on anionic transporters have been observed in probenecid. Therefore, probenecid has biological effects other than its major role as ULT. Probenecid has neuroprotective effects via inhibiting organic anion transporters in Huntington’s disease [22]. Its anti-hypertensive effects occur via α1-adrenergic receptor inhibition [23]. However, no significant anti-hypertensive effect was observed in our study. Probenecid can protect against transient global cerebral ischemia and reperfusion injury by inhibiting the calpain–cathepsin pathway [24].

Based on a model involving lipotoxic liver injury, the abnormal activation of pannexin 1 can induce hepatic inflammation, while probenecid may inhibit pannexin 1 channels [25, 26]. An elevated uric acid level is one danger signal in alcoholic steatohepatitis [27]. During initial gout flares, aggressive control of hyperuricemia may decrease liver damage. In this clinical trial, the possible effect of liver protection was also observed. No significant elevation of liver function was found in the group that received a combination of probenecid and colchicine compared to the group that received monotherapy with colchicine. However, the sample size was small in this study, and this finding of liver protection requires further evaluation.

Overall, the mean pain severity was similar between groups throughout the 15 days of treatment (Fig. 3). However, there was a significant decrease in serum uric acid levels in the treatment group after 15 days of medication (Fig. 4). The average pain duration was also similar between the two groups (Fig. 5). Early and aggressive control hyperuricemia by probenecid with colchicine could not induce higher pain severity and duration in our study. In the other study, the chronic GA patients with a serum CRP level > 3 mg/dL who did not take prophylaxis were associated with gout flare recurrence during initial ULT therapy [28].

Low-dose colchicine (0.5 mg once or twice daily) or low-dose NSAIDs can serve as prophylactic treatment during ULT in GA [29, 30]. In our study, colchicine was administered with intermittent NSAIDs and no significant elevation in gout flares was found. Adequate control of hyperuricemia and reducing tophus size are associated with a lower probability of relapse during ULT [31]. Therefore, our study findings support the concept of early ULT in gout under adequate prophylactic treatment.

The limitations of this study were as follows: first, it enrolled a small number of participants; second, pain was evaluated using VAS scores without serum markers; third, the serum observation time was relatively short; and fourth, intermittent NSAID usage may have introduced bias due to pain control.

## Conclusion

In summary, no significant increase was noted in acute gout flare duration or severity among patients with aggressive control of hyperuricemia using probenecid in our study. Our findings suggest the efficacy of early and aggressive lowering of serum uric acid levels using ULT with combination of prophylactic treatment.

## Data Availability

The data of this study were collected from Taichung Armed forces General Hospital and Tri-Service General Hospital in Taiwan. Partial contents of this paper were released as an oral presentation with an abstract (SU058) at the 2018 Asia-Pacific League of Associations for Rheumatology Congress (International Journal of Rheumatic Disease; https://doi.org/10.1111/1756-185X.13361).
